# Oncolytic Reovirus in Canine Mast Cell Tumor

**DOI:** 10.1371/journal.pone.0073555

**Published:** 2013-09-20

**Authors:** Chung Chew Hwang, Saori Umeki, Masahito Kubo, Toshiharu Hayashi, Hiroshi Shimoda, Masami Mochizuki, Ken Maeda, Kenji Baba, Hiroko Hiraoka, Matt Coffey, Masaru Okuda, Takuya Mizuno

**Affiliations:** 1 Laboratory of Veterinary Internal Medicine, The United Graduate School of Veterinary Science, Yamaguchi University, Yamaguchi, Japan; 2 Laboratory of Veterinary Pathology, Joint Faculty of Veterinary Medicine, Yamaguchi University, Yamaguchi, Japan; 3 Laboratory of Veterinary Microbiology, Joint Faculty of Veterinary Medicine, Yamaguchi University, Yamaguchi, Japan; 4 Emerging Infectious Diseases, Joint Faculty of Veterinary Medicine, Kagoshima University, Kagoshima, Japan; 5 Yamaguchi University Animal Medical Center, Yamaguchi, Japan; 6 Oncolytics Biotech Inc., Calgary, Alberta, Canada; 7 Laboratory of Veterinary Internal Medicine, Joint Faculty of Veterinary Medicine, Yamaguchi University, Yamaguchi, Japan; 8 Biomedical Science Center for Translational Research, The United Graduate School of Veterinary Science, Yamaguchi University, Yamaguchi, Japan; McMaster University, Canada

## Abstract

The usage of reovirus has reached phase II and III clinical trials in human cancers. However, this is the first study to report the oncolytic effects of reovirus in veterinary oncology, focusing on canine mast cell tumor (MCT), the most common cutaneous tumor in dogs. As human and canine cancers share many similarities, we hypothesized that the oncolytic effects of reovirus can be exploited in canine cancers. The objective of this study was to determine the oncolytic effects of reovirus in canine MCT *in vitro*, *in vivo* and *ex vivo*. We demonstrated that MCT cell lines were highly susceptible to reovirus as indicated by marked cell death, high production of progeny virus and virus replication. Reovirus induced apoptosis in the canine MCT cell lines with no correlation to their Ras activation status. *In vivo* studies were conducted using unilateral and bilateral subcutaneous MCT xenograft models with a single intratumoral reovirus treatment and apparent reduction of tumor mass was exhibited. Furthermore, cell death was induced by reovirus in primary canine MCT samples *in vitro*. However, canine and murine bone marrow-derived mast cells (BMCMC) were also susceptible to reovirus. The combination of these results supports the potential value of reovirus as a therapy in canine MCT but warrants further investigation on the determinants of reovirus susceptibility.

## Introduction

Cancer is the leading cause of human death in developed countries and this number is projected to rise each year [1]. Even though precise estimates of global cancer morbidity and mortality rates in pets are unavailable due to the lack of census, cancer has also been ascertained to be a major cause of death in pets. Furthermore, there has been an upward trend in the number of pets dying of cancer, partly due to pets living to an increasingly older age and improved veterinary care [2]. Therefore, there is a dire need for more advanced and effective animal cancer therapy.

Over the last decade, one of the most interesting oncotherapies to have emerged is oncolytic virotherapy. Adenoviruses, herpes simplex viruses, vaccinia virus and reovirus are among the viruses that have shown promising results, up to the extent of phase II and III clinical trials in human cancers, including cancer of the liver, pancreas, prostate, ovaries, head and neck, glioma, melanoma and other solid tumors [3]. However, the use of oncolytic virotherapy in veterinary medicine is still far from becoming commercially available as promising laboratory results have yet to be translated into clinical outcomes. So far, canine cancers, such as osteosarcoma, malignant melanoma, lymphoma, soft tissue sarcoma, mammary adenoma and carcinoma, have been tested with only a few oncolytic viruses, mainly the human and canine adenoviruses, canine distemper virus (CDV) and vaccinia virus strains [4].

Reovirus, a small non-enveloped icosahedral virus with 10 segments of double stranded RNA, can be found ubiquitously and reovirus seropositivity is high among adults even though they may be asymptomatic [5]. Among the 3 reovirus serotypes, the potential of the serotype 3 Dearing strain has been extensively studied in a wide range of cancers [6]–[8]. Despite its popularity in human cancers, the effects of reovirus have not been tested in any naturally occurring animal cancer so far. Reovirus was first discovered as a naturally occurring replication-competent oncolytic virus when it infected and killed transformed cells but left normal cells unharmed [9]. Transformation of cell lines that were naturally resistant to reovirus with v-*erbB*, *sos* or *ras* (all activators of Ras signaling pathway) allowed the cells to be highly susceptible to reovirus infection [10], [11]. Phosphorylation of the PKR (dsRNA-activated protein kinase) has been identified to be one of the major factors that inhibited the translation of viral genes and viral replication in untransformed cells [11], [12]. While Ras activation has been proven to enhance reolysis, further research has shown that reovirus can exert its oncolytic effects independent of this pathway [13]. This highlights the complexity of the mechanism in which reovirus works in cancer cells and that our current understanding is insufficient to pinpoint a definitive biomarker of susceptibility to reovirus.

Mast cell cancer is rare in humans [14] but mast cell tumor (MCT) is the most common cutaneous tumor in dogs, comprising approximately 16% to 21% of all canine cutaneous tumors [15]. Complete surgical excision is potentially curative in well-differentiated and intermediate grade canine MCT while radiation or medical therapy is often necessary as adjunctive therapy for incompletely resected tumors. However, undifferentiated canine MCT is an aggressive tumor that frequently metastasizes to local lymph nodes, spleen, liver, and possibly to the bone marrow and peripheral blood. Most dogs with the aggressive form of the tumor die within one year of diagnosis. Therefore, new therapeutic approaches to canine MCT are needed.

Despite the fact that mutation in *ras* itself is uncommon in canine cancers [16], [17], we hypothesized that canine cancers are susceptible to reovirus as naturally occurring cancers of dogs and humans have many similarities [18]. In this study, we examined the oncolytic effects of reovirus in canine MCT *in vitro*, *in vivo* and *ex vivo*. We also examined the relationship between reovirus susceptibility and the Ras activation status in the MCT cell lines. To the best of our knowledge, this is the first study on the oncolytic effects of reovirus in veterinary oncology.

## Materials and Methods

### Ethics statement

All animal procedures were conducted in accordance to the Yamaguchi University Animal Care and Use guidelines and were approved by the Institutional Animal Care and Use Committee of Yamaguchi University (Permit Number: 188). All efforts were made to ensure minimal pain and suffering of the animals.

### Cell cultures and reovirus

Four established canine MCT cell lines, VIMC, CoMS, CM-MC and HRMC, were used in this study. VIMC [19] and CoMS [20] were derived from visceral MCT while CM-MC [19] and HRMC [21] were MCT of cutaneous origin. RBL-2H3, a rat basophilic leukemia cell line, and P815, a mouse lymphoblast-like mastocytoma cell line, were selected as comparison models of mast cell across species while the human Burkitt's lymphoma cell lines, Daudi and Raji, were used as negative and positive controls respectively [6]. Mouse L929 fibroblastic cell line was used in the titration of progeny virus. RBL-2H3, P815, Raji and L929 were obtained from the Cell Resource Center for Biomedical Research (Institute of Development, Aging and Cancer, Tohoku University, Sendai, Japan). All cells were maintained in R10 complete medium (RPMI1640 supplemented with 10% FBS, 100 U/ml penicillin, 100 μg/ml streptomycin and 55 μM 2-mercaptoethanol) kept at 37°C in a humidified 5% CO_2_ incubator.

The Dearing strain of reovirus serotype 3 (Reolysin®; clinical grade reovirus; GMP) was obtained from Oncolytics^TM^ Biotech Inc. (Calgary, Canada) through a collaborative effort. Ultraviolet (UV)-inactivated virus was prepared by exposing live virus to UV light for 90 minutes.

### Reovirus infection of cell lines

VIMC, CoMS, CM-MC, HRMC, Daudi and Raji were seeded at 2.5×10^4^ cells while RBL-2H3 and P815 were seeded at 1.25×10^4^ cells before being mock-infected or infected with reovirus at multiplicity of infection (MOI) of 70 plaque-forming units (PFUs) per cell. All cells were seeded in triplicates in 48-well plates. Cells were stained with 0.25% trypan blue and viability counted via hemocytometer at 72 hours post-infection (hpi). Supernatant from each sample was collected and kept at −80°C, pending titration of progeny virus using 50% tissue culture infectious dose (TCID_50_) assay on L929 cells, as previously described [22] with modifications.

The same cell number of canine MCT cells was also used to assess the cell viability curve at 0, 24, 48 and 72 hpi at MOI 70 of reovirus. To assess the sensitivity of cells towards various titer of reovirus, the same cell number of canine MCT cells was incubated with UV-inactivated, 2.8, 14 and 70 MOI of reovirus for 72 hours before cell viability and titer of progeny virus were assessed.

### Reovirus infectivity and Poly (ADP-Ribose) Polymerase cleavage (PARP)

Canine MCT cells were seeded at 5.0×10^5^ and mock-infected or infected with reovirus at MOI 70 for 6 and 24 hours. The same number of control cells (Daudi and Raji) was mock-infected or infected with reovirus at MOI 70 and cells were harvested at 6, 24 and 48 hpi. Whole cell lysates for reovirus infectivity were treated with RIPA lysis buffer (50 mM Tris (pH7.5), 150 mM NaCl, 1% NP40, 0.5% sodium deoxycholate) while lysates for PARP cleavage were lysed with NP40 lysis buffer (1% NP40, 10 mM Tris HCl (pH 7.5), 150 mM NaCl, 1 mM EDTA). Proteins were subjected to SDS-PAGE and Western blotting.

### Measurement of cell death by propidium iodide (PI) staining

Canine MCT cells that were seeded at 1.0×10^5^ were infected with reovirus at MOI 70 and cells were harvested at 48 and 72 hpi. Cells were washed with cold PBS and fixed with cold ethanol before storage at −20°C, pending analysis. Cells were incubated with 100 μl of 100 μg/ml Ribonuclease A (Nalacai Tesque, Kyoto, Japan) for 30 minutes before 4 μl of 1 mg/ml PI (Sigma-Aldrich Japan K.K., Tokyo, Japan) was added 5 minutes before acquisition. Flow cytometry was performed using CyFlow® Space (Partec GmbH, Münster, Germany) and results were analyzed using FlowJo software (Tree Star, Inc., San Carlos, CA, USA). The percentage of subG1 cells were considered as apoptotic cells.

### Inhibition of reovirus cell killing by Z-VAD-FMK

To provide further evidence that reovirus-induced cell death was due to apoptosis, 2.5×10^4^ cells were seeded in triplicate and pre-treated with control DMSO, 10 μM or 100 μM of Z-VAD-FMK (caspase inhibitor I; Calbiochem, Billerica, MA, USA) for 30 minutes at 37°C before being infected with reovirus at MOI 70. Viable and non-viable cells were assessed using 0.25% trypan blue at 48 hpi.

### GST pull-down assay for Ras status

Ras activation status of each cell line was evaluated after GST pull-down [23]. To construct a vector that expresses GST fused with Ras-binding domain (RBD) of Raf-1, a part of Raf-1 gene encoding amino acids 1−149 of Raf-1 was amplified using plasmid pCMV5 Raf-1 as a template and primers YTM647 (5′ CGTGGA TCCGAGCACATACAGGGAGCT 3′, underline indicates the BamHI site) and YTM648 (5′ CGGGAATTCAGCTTCAGGAACGTCTT 3′, underline indicates the EcoRI site) as previously described [24]. The amplified PCR products were subcloned into the BamHI and EcoRI sites of the pGEX 4T-3 vector (pGEX-RBD#2). JM109 was transformed with pGEX-RBD#2 and GST-RBD was extracted with lysis buffer. Cytoplasmic extract from cells (300 μg) was mixed with glutathione-Sepharose 4B beads (GE Healthcare, Tokyo, Japan) conjugated with GST-RBD protein for 1 hour before washing with lysis buffer. Precipitated Ras-GTP and whole cell lysates were subjected to SDS-PAGE, followed by Western blotting.

### Western blotting

Following electrophoresis, proteins were transferred to polyvinylidene fluoride (PVDF) membranes and probed with specific primary antibodies as follows: rabbit anti-reovirus (produced by our lab), rabbit anti-PARP (NeoMarkers, Fremont, CA, USA) or mouse anti-pan-Ras (Calbiochem). Incubation with primary antibodies was followed by secondary labeling using goat anti-rabbit or goat anti-mouse IgG HRP (Zymed Laboratories, San Francisco, CA, USA). The membranes were visualized by immersion in Western Lightning Chemiluminescence reagent (PerkinElmer, Shelton, CT, USA). Immunoreactive bands were visualized using the Luminescent Image Analyzer LAS 3000 mini (FUJIFILM, Tokyo, Japan) and analyzed using Science Lab 2005 (FUJIFILM). Membranes were stripped between antibody staining procedures with stripping buffer (100 mM 2-mercaptoethanol, 2% SDS, 62.5 mM Tris (pH6.7)) for 30 minutes at 60°C. Goat anti-actin (Santa Cruz Biotechnology, Inc., Santa Cruz, CA, USA) and rabbit anti-goat IgG HRP (Bethyl Laboratories, Inc., Montgomery, TX, USA) were used as loading controls.

### Subcutaneous tumor xenograft models in NOD/SCID mice

Eight to nine-week-old NOD/ShiJic-*scid* (NOD/SCID) mice were obtained from Kyudo Co. Ltd. (Saga, Japan) and studies were conducted in a specific pathogen-free area in accordance with the Yamaguchi University Animal Care and Use guidelines. VIMC or CoMS cells (1.0×10^7^ in 50 μl PBS) were implanted subcutaneously into one or both flanks of the mice under general anesthesia. When the desirable tumor size was achieved on either side, 7.0×10^7^ PFUs of live reovirus (experimental group) or UV-inactivated reovirus (control group) in 20 μl PBS were injected intratumorally. Two-dimensional tumor measurements were performed with a caliper every other day until euthanasia due to excessive tumor burden. Tumor measurements were analyzed and shown as tumor mass (mm^3^). Tumors and remaining masses were fixed in 10% neutral buffered formalin and embedded in paraffin before staining with hematoxylin and eosin (H&E) for histopathological analysis.

For immunohistochemical (IHC) staining, deparafinized samples were treated with Target Retrieval Solution (Dako, Glostrup, Denmark) before treatment with 3% hydrogen peroxidase and Protein Block (Dako). Sections were then incubated with rabbit anti-reovirus polyclonal antibody (1∶500 dilution; produced by our lab), followed by Histofine Simple Stain MAX-PO (R) (Nichirei Biosciences, Inc., Tokyo, Japan). Slides were subjected to 3,3-diaminobenzidine tetrachloride (Roche Diagnostics K.K., Tokyo, Japan) staining before counterstaining with Meyer's hematoxylin.

### Reovirus infection of primary canine MCT samples

Primary canine MCT tumor cells were obtained by fine needle aspiration (FNA) from canine patients with confirmed diagnosis of MCT at the Yamaguchi University Animal Medical Center. Immediately after collection, 2.5×10^4^ cells were seeded in triplicate before being mock-infected or infected with reovirus at MOI 70. Viability of cells was assessed at 72 hpi with 0.25% trypan blue and progeny virus was measured using TCID_50_ assay.

### Generation of canine and murine bone marrow-derived cultured mast cells (BMCMC)

Canine BMCMC was isolated and purified from bone marrow cells of two healthy beagles as previously reported [25], [26]. Briefly, bone marrow cells were separated using Lymphoprep™ kit (Axis-Shield PoC AS, Oslo, Norway) before CD34^+^ cells were enriched using MidiMACS (Miltenyi, Auburn, CA, USA) separator. CD34^+^ cells were cultured in Stemline II (Sigma-Aldrich) supplemented with 100 ng/ml recombinant canine stem cell factor (rcSCF; R&D Systems, Minneapolis, MN, USA) and incubated at 37°C in a humidified 5% CO_2_ incubator. Matured CD117^+^ cells were enriched with MidiMACS separator again 4 weeks after culture.

Murine BMCMC was generated from bone marrow cells of C57BL/6N mice (Kyudo Co. Ltd.) and cultured in D10 (DMEM with 10% FBS, 100 U/ml penicillin, 100 μg/ml streptomycin and 55 μM 2-mercaptoethanol) supplemented with murine IL-3 (PeproTech Inc., Rocky Hill, NJ, USA) [27]. Cultures were grown at 37°C in a humidified 5% CO_2_ incubator for 5 weeks before CD117^+^ cells were enriched with MidiMACS separator.

Purity of canine and murine BMCMC were confirmed with toluidine blue staining after attaching BMCMC on glass slides using cytospin and fixing in Carnoy's solution for 30 minutes. Both canine and murine BMCMC were seeded in triplicate before being mock-infected or infected with reovirus at MOI 70 and cell viability was assessed at 72 hpi with 0.25% trypan blue. Progeny virus produced was titered using TCID_50_ assay.

## Results

### MCT cell lines are susceptible to reovirus-induced cytotoxicity *in vitro*

**Figure 1:**
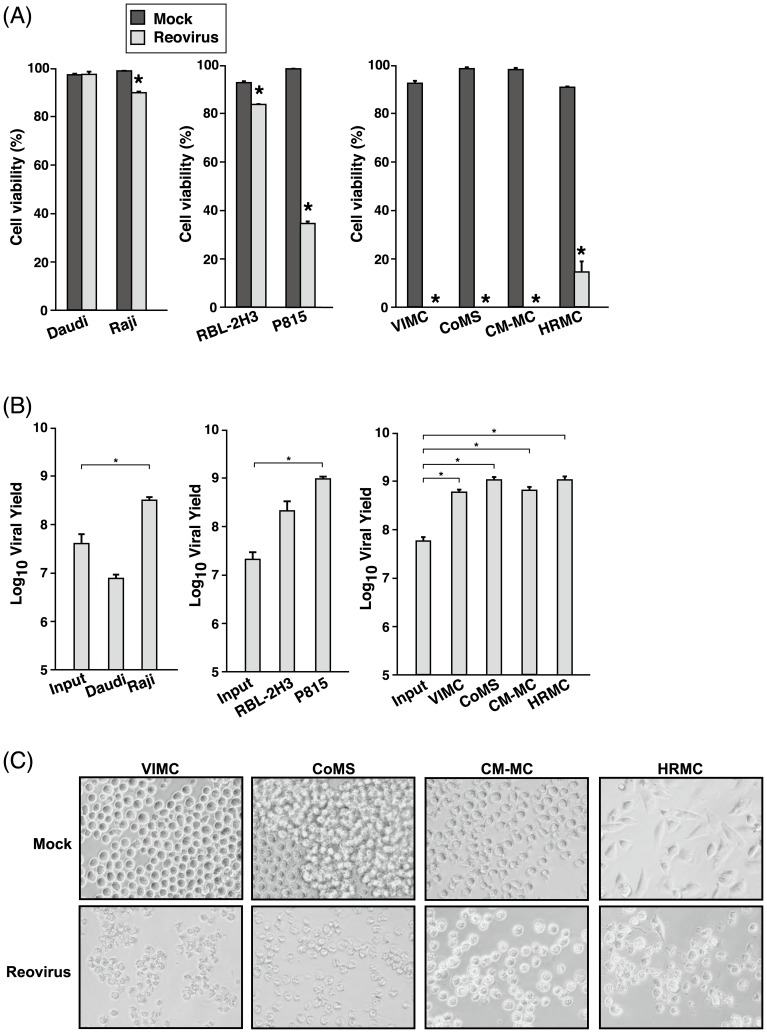
Reovirus effectively exerts effect in MCT cell lines. (A) VIMC, CoMS, CM-MC, HRMC, RBL-2H3 and P815 cells in triplicate wells were either mock-infected or infected with reovirus at MOI 70. Human Burkitt's lymphoma cell lines, Daudi and Raji, were used as negative and positive controls respectively. After 72 hours post-infection (hpi), cell viability was assessed with 0.25% trypan blue. Data represents the mean and standard deviation of three independent experiments. *, p<0.05 (by Student's *t* test). (B) Supernatant of reovirus-infected (MOI 70) MCT cell lines was harvested at 72 hpi before input and progeny virus titer were determined by TCID_50_ assay. Data represents the log_10_ mean viral yield and standard deviation from three independent experiments. *, p<0.05 (by Student's *t* test). (C) Photomicrographs of mock-infected (upper panels) and reovirus-infected (MOI 70; lower panels) canine MCT cells taken at 72 hpi.

The susceptibility of MCT cell lines to reovirus infection and oncolysis was first examined with MOI 70 of reovirus and cell viability was assessed at 72 hpi. All MCT cell lines, especially the canine MCT cell lines, were susceptible to reovirus (Fig. 1A; p<0.05). At 72 hpi, 100% cell death was induced in VIMC, CoMS and CM-MC and more than 80% was induced in HRMC. Reovirus-infected P815 showed a higher percentage of cell death in comparison to RBL-2H3. Cell viability was also reduced (p<0.05) in Raji, but not in Daudi.

Following reovirus infection, P815, VIMC, CoMS, CM-MC, HRMC and Raji produced a significant amount of progeny virus at 72 hpi, while Daudi did not (Fig. 1B). This indicates that reovirus is able to replicate in susceptible cell lines and infectious progeny virus is released for subsequent infection cycles. Even though progeny virus was produced in RBL-2H3, the amount was not significantly higher than the input titer. Examination of the morphology of the canine MCT cell lines revealed distinct virus-induced cytopathic effects (CPE) at 72 hpi in contrast to the mock-infected cells (Fig. 1C).

### Reovirus is highly infective in canine MCT cell lines

**Figure 2:**
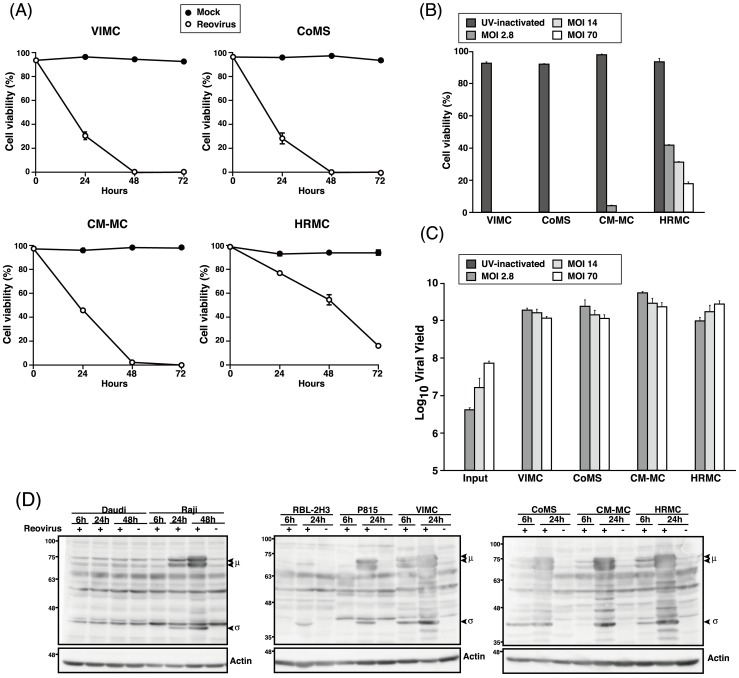
Reovirus infectivity and replication in MCT cell lines. (A) Cell viability of reovirus-infected (MOI 70) canine MCT cell lines were assessed with 0.25% trypan blue at 0, 24, 48 and 72 hpi. (B) Canine MCT cell lines were treated with UV-inactivated, 2.8, 14 and 70 MOI of reovirus and cell viability was assessed with 0.25% trypan blue at 72 hpi. (C) Input and progeny virus titer of supernatant from reovirus-infected MCT cell lines at various MOI were determined by TCID_50_ assay. Data represents the mean and standard deviation of three independent experiments. (D) Whole cell lysates of reovirus-infected (MOI 70) of cell lines at 6 and 24 hpi (48 hpi only for Daudi and Raji) were prepared before proteins were separated using SDS-PAGE electrophoresis. Presence of μ and σ outer capsid proteins was determined using rabbit polyclonal anti-reovirus antibody. β-actin was used as protein loading controls.

The kinetics of reovirus infection in canine MCT cell lines at 0, 24, 48 and 72 hpi at MOI 70 was assessed. By 48 hpi, 100% cell death was seen in VIMC and CoMS while more than 95% and 45% cell death was detected in CM-MC and HRMC (Fig. 2A). This indicates that canine MCT cell lines were highly susceptible to reovirus, with VIMC and CoMS being the most susceptible, followed by CM-MC and HRMC.

When the canine MCT cell lines were inoculated with UV-inactivated, 2.8, 14 and 70 MOI of reovirus, 100% cell death in VIMC and CoMS was induced at MOI as low as 2.8 PFUs/ cell. At the same MOI, more than 95% and 50% cell death was induced in CM-MC and HRMC respectively (Fig. 2B). Measurement of the titer of progeny virus produced in the cell lines revealed that the titer was comparable among the various groups of MOI, suggesting that the amount of virus used in the initial infection does not play a critical role in inducing cytolysis in reovirus susceptible MCT cell lines (Fig. 2C). No progeny virus was detected in the UV-treated group.

To confirm the infectivity of canine MCT cell lines to reovirus, detection of the reovirus μ and σ protein was carried out via Western blotting. The synthesis of both the μ and σ protein was detected as early as 6 hpi in all the canine MCT cell lines as compared to P815, RBL-2H3 and Raji (Fig. 2D), suggesting that reovirus replicates more efficiently in canine MCT cell lines. Synthesis of reovirus protein was not detected in Daudi even up to 48 hpi, confirming that Daudi is resistant to reovirus.

### Reovirus induces apoptosis in MCT cell lines

**Figure 3:**
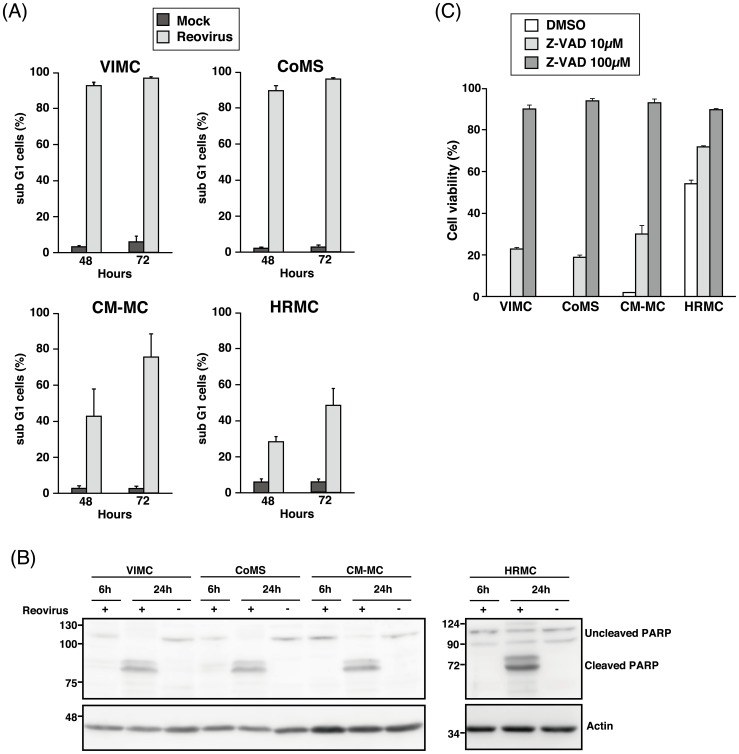
Reovirus induces cell death in MCT cell lines via the apoptosis pathway. (A) Reovirus-infected (MOI 70) canine MCT cell lines were harvested at 48 or 72 hpi and stained with propidium iodide (PI) before fluorescence-activated cell sorting (FACS) acquisition. Cell death was determined by the proportion of subG1 cells. Data represents the mean and standard deviation of three independent experiments. (B) Whole cell lysates of reovirus-infected (MOI 70) canine MCT cell lines at 6 and 24 hpi were prepared before proteins were separated using SDS-PAGE electrophoresis. Presence of Poly(ADP-Ribose) Polymerase (PARP) cleavage was determined using anti-PARP antibody. β-actin was used as protein loading controls. (C) Canine MCT cell lines in triplicate wells were pre-treated with DMSO, 10 μM or 100 μM of Z-VAD-FMK for 30 minutes at 37°C incubator before being infected with reovirus at MOI 70. Cell viability was assessed with 0.25% trypan blue at 48 hpi. Data represents the mean and standard deviation of three independent experiments.

Confirmation of cell death in canine MCT cell lines was subsequently assessed with PI staining. An increment in the proportion of the subG1 cells indicated that the number of apoptotic cells increased from 48 to 72 hpi. At 72 hpi, more than 95% cell death was seen in VIMC and CoMS, 75% in CM-MC and 48% in HRMC (Fig. 3A). The consistency of these results indicates that although trypan blue is usually used as a fast and easy screening of cell viability, it is a reliable technique to directly display the proportion of viable and non-viable cells.

Western blot analysis of PARP cleavage in the canine MCT cell lines confirmed the appearance of the signature cleavage product as early as 6 hpi in VIMC and CoMS. Cleaved PARP was visualized in all the canine MCT cell lines at 24 hpi as compared to the mock-infected samples (Fig. 3B). The cleavage of cellular substrate PARP in reovirus-infected cells demonstrated the morphological hallmark of apoptosis.

Canine MCT cell lines were also pre-treated with Z-VAD-FMK before infection with reovirus and cell viability was determined at 48 hpi. Reovirus-induced cytotoxicity was reversed by Z-VAD-FMK in all the cell lines where cell death was slightly inhibited at 10 μM and almost completely inhibited at 100 μM (Fig. 3C). The combination of these results allows us to conclude that reovirus induces canine MCT cell death predominantly through an apoptotic pathway.

### Ras is not activated in MCT cell lines

**Figure 4:**
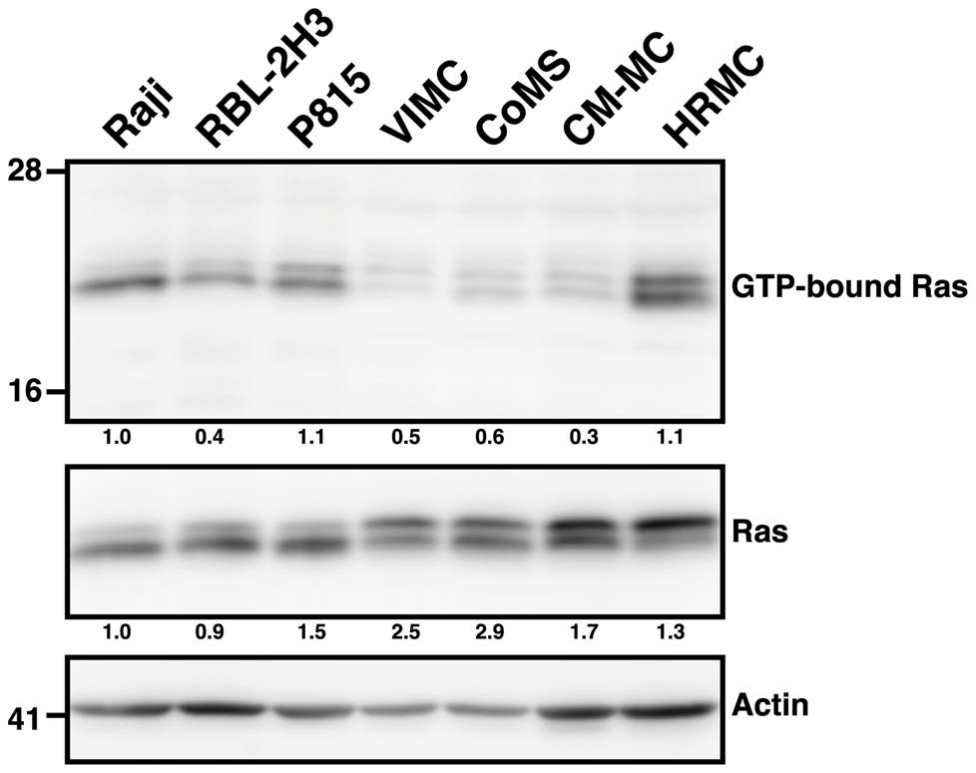
Ras activation does not correlate with reovirus susceptibility in MCT cell lines. GST-RBD protein was extracted and immobilized on glutathione-Sepharose beads to affinity-precipitated Ras-GTP from cell lysates. The affinity-precipitated Ras-GTP and whole cell lysates were subjected to SDS-PAGE before detection by Western blotting with anti-Ras Ab. The number below each lane represents the densitometry analysis of GTP-bound Ras relative to actin (upper row) and total Ras relative to actin (lower row) using ImageJ. Raji was used as the standard for Ras activation.

Ras activation has been reported to contribute to proteolytic reovirus disassembly as well as increased progeny virus infectivity and release [28]. Since reovirus has not been tested in any canine cancer, it remains unclear if the Ras signaling pathway plays a role in reovirus susceptibility in these cells. Using Raji as the standard of Ras activation, our results showed that P815 and HRMC have elevated Ras activity, while RBL-2H3, VIMC, CoMS and CM-MC did not (Fig. 4). Even though all of the MCT cell lines were susceptible to reovirus, the level of activated Ras expression varied. The notion that reovirus susceptibility is dependent on the Ras activation status is inconsistent, especially when compared to the three canine MCT cell lines that are the most susceptible to the effects of reovirus. This finding indicates that reovirus exerts its potent oncolytic effects in MCT cell lines independent of the Ras signaling pathway.

### Reovirus induces regression of MCT mass *in vivo*

**Figure 5:**
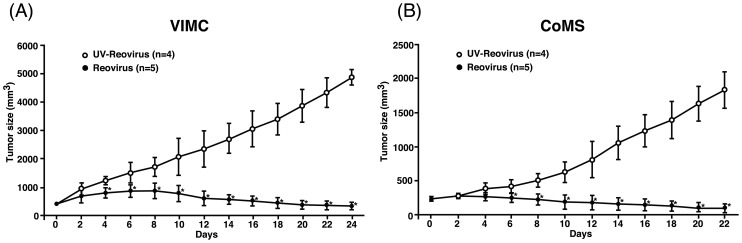
Reovirus effectively reduces tumor mass in unilateral MCT xenograft mouse models. (A) VIMC and (B) CoMS (1×10^7^ cells) were implanted subcutaneously at the right flank. Once the desired tumor size was achieved (day 0), tumors were treated with a single intratumoral injection of 7×10^7^ PFUs of reovirus or UV-inactivated reovirus. Tumor size was measured every other day with a caliper. Data represents the mean and standard deviation of each treatment group. *, p<0.05 (by Student's *t* test).

To assess the therapeutic potential of reovirus *in vivo*, VIMC and CoMS unilateral subcutaneous xenograft models were established in NOD/SCID mice and treated with a single intratumoral of reovirus injection. All mice treated with reovirus experienced significant regression of tumor mass by day 4 post-treatment for VIMC and by day 6 post-treatment for CoMS (Fig. 5A and 5B; p<0.05). However, black tail syndrome, a possible side effect in reovirus-infected NOD/SCID mice [29], was observed in one out of five in VIMC and three out of five in CoMS-transplanted mice that were treated with reovirus.

**Figure 6:**
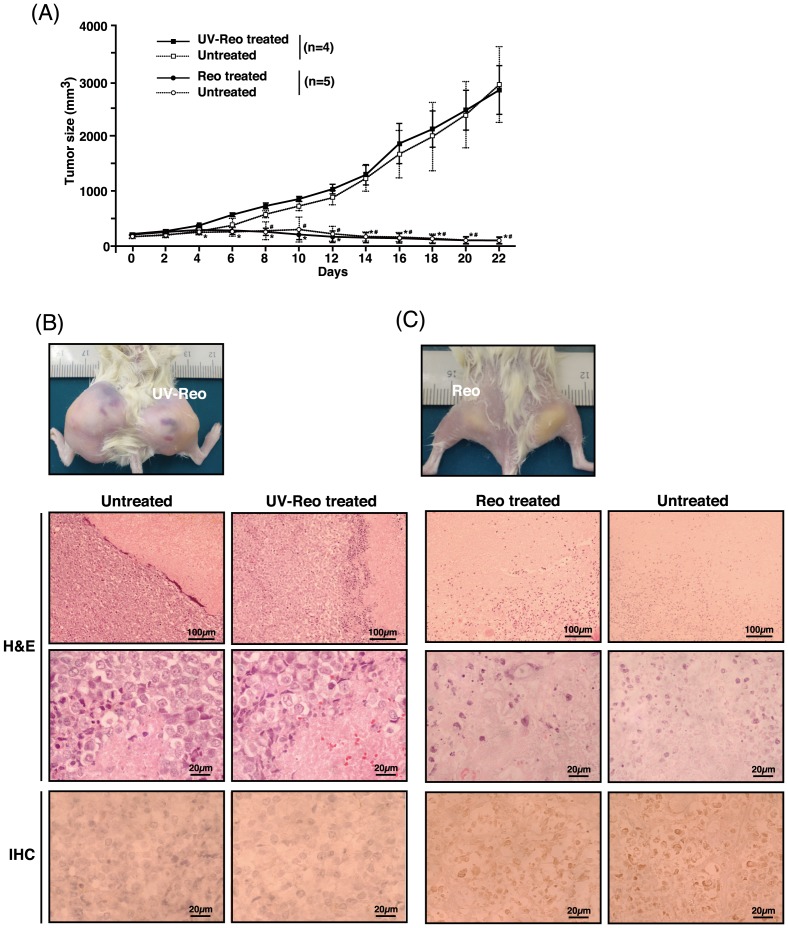
Gross and histological appearance of VIMC bilateral MCT xenograft mouse models. (A) VIMC (1×10^7^ cells) was implanted subcutaneously into both flanks. When either one of the tumors reached the desired size (day 0), tumors were treated unilaterally with a single intratumoral injection of 7×10^7^ PFUs of reovirus or UV-inactivated reovirus. Tumor size was measured every other day with a caliper. Data represents the mean and standard deviation of each treatment group. *, p<0.05 between reovirus and UV-inactivated reovirus treated masses; #, p<0.05 between the contralateral masses. Gross appearance, H&E and IHC histological samples of representative bilateral VIMC mice 22 days after treatment with UV-inactivated reovirus (B) or reovirus (C).

Anticipating that reovirus-susceptible tumor would allow generation of adequate progeny virus for hematogenous dissemination, bilateral cutaneous VIMC xenograft models were created and treated with a single unilateral intratumoral reovirus injection on either mass. As expected, significant tumor regression was not only limited to the intratumoral-treated tumor, but was also observed in the non-treated contralateral tumor (Fig. 6A; p<0.05). This suggests that a substantial amount of reovirus was produced and released from the treated tumor into the blood circulation. No side effects were observed in this group of mice.

In contrast to the UV-inactivated reovirus treated tumors (Fig. 6B), H&E-stained histopathological samples from the bilateral VIMC xenograft models showed extensive necrotic lesions within reovirus-treated tumors (Fig. 6C), confirming the presence of anti-cancer activity. Similar extensive necrotic lesions were also seen in the histopathological samples of the unilateral xenograft models (data not shown). IHC staining demonstrated the presence of reovirus proteins only in the reovirus-treated and the contralateral tumors (Fig. 6C).

### Primary canine MCT, normal canine and murine BMCMC are susceptible to reovirus

**Figure 7:**
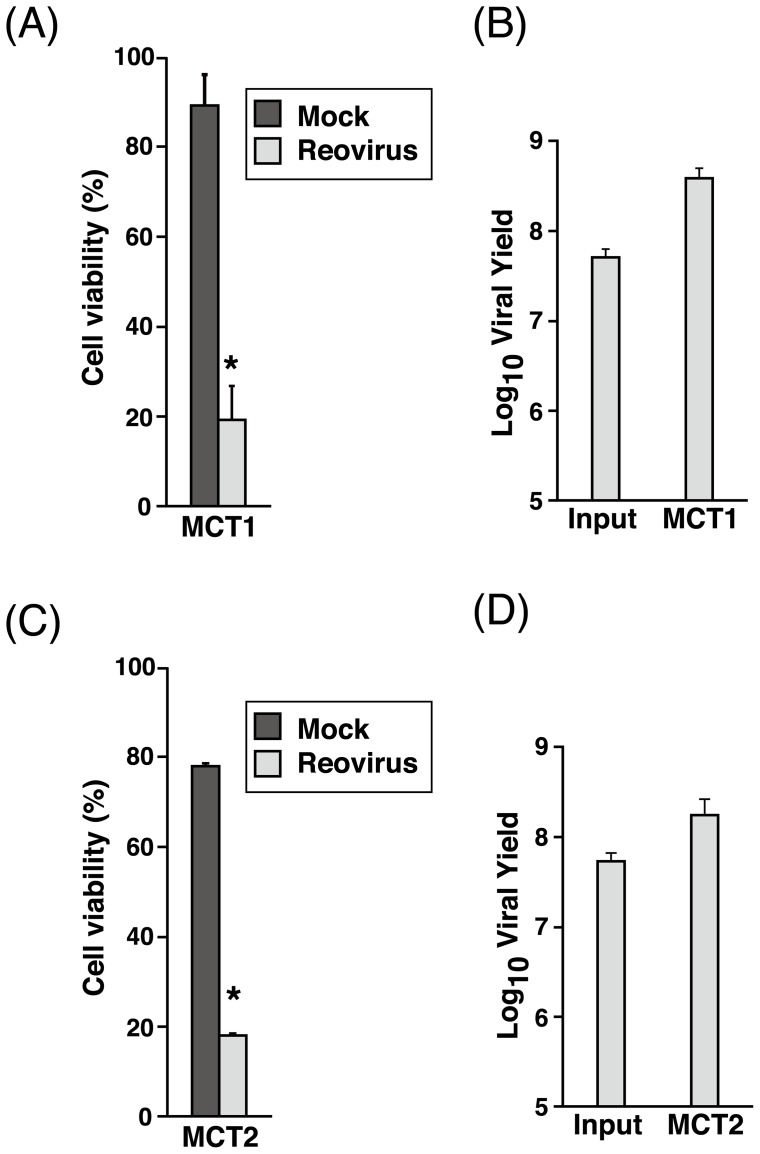
Reovirus induces significant cell death in primary canine MCT samples. Primary canine MCT samples in triplicate wells were either mock-infected or infected with reovirus at MOI 70. Cell viability was assessed with 0.25% trypan blue at 72 hpi before input and progeny virus titer were determined by TCID_50_ assay. Data from MCT 1 (A, B) represents the mean and standard deviation three independent experiments while two independent experiments were conducted for MCT 2 (C, D). *, p<0.05 (by Student's *t* test).

The next step of our study was to determine reovirus oncolysis in primary canine MCT samples. Reovirus induced approximately 80% cell death in both the primary MCT samples tested and an increased amount of progeny virus was produced (Fig. 7). This strongly supports the feasibility of using reovirus as a potential therapeutic option in cases of canine MCT.

**Figure 8:**
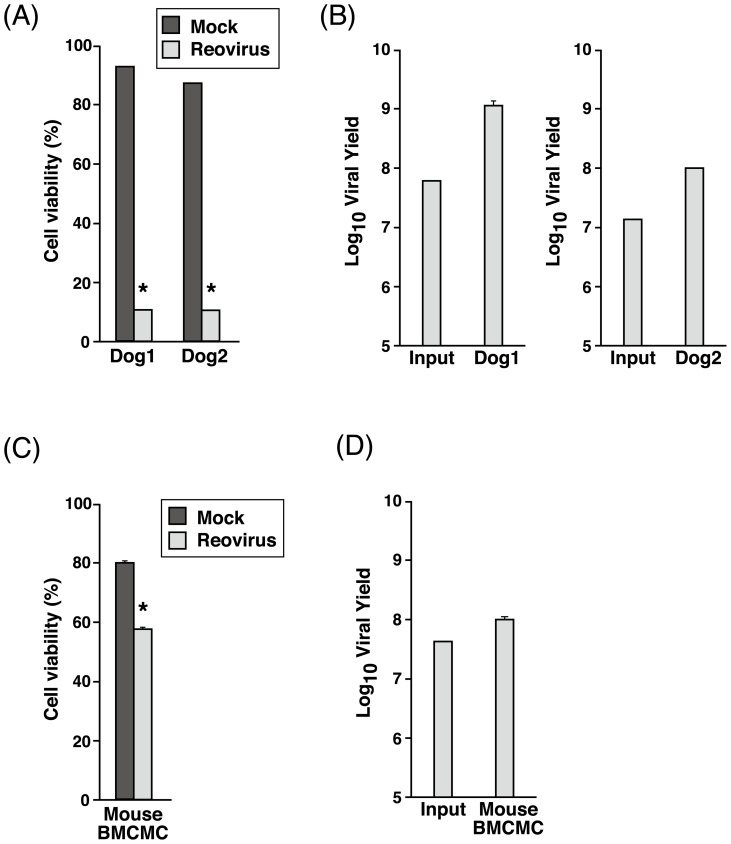
Reovirus induces dramatic effects in canine BMCMC but milder effects in murine BMCMC. Canine and murine BMCMC in triplicate wells were either mock-infected or infected with reovirus at MOI 70 before cell viability was examined with 0.25% trypan blue at 72 hpi before input and progeny virus titer were determined using TCID_50_ assay. Mean and standard deviation from dog 1 and 2 were shown in (A, B) while data of mouse BMCMC (C, D) represents the mean and standard deviation from three C57BL/6N mice. *, p<0.05 (by Student's *t* test).

To assess the effects of reovirus in normal mast cells, canine and murine BMCMC were generated and infected with reovirus. Cell viability was markedly reduced in the canine BMCMC (∼90% cell death) (Fig. 8A) as compared to the murine BMCMC (∼40% cell death) (Fig. 8C). Consequently, reovirus-infected canine BMCMC produced a higher titer of progeny virus as compared to murine BMCMC (Figs. 8B and 8D), suggesting that even normal mast cells are able to support reovirus replication. These results show that reovirus does not have an infectivity preference towards normal healthy or abnormal tumorous mast cells.

## Discussion

Reovirus has been extensively studied in human oncology over the past decade. Due to the relative similarities between human and canine cancers, we hypothesized that the cytotoxic effects of reovirus could be exploited in canine cancers as well. To our knowledge, this is the first report of the effects of reovirus in veterinary oncology, where the susceptibility of canine MCT *in vitro*, *in vivo* and *ex vivo* towards the oncolytic effects of reovirus was determined. We demonstrated that MCT cell lines are susceptible to reovirus, especially in canine MCT. Moreover, these *in vitro* findings were confirmed *in vivo*, whereby LuMC and CoMS subcutaneous tumors underwent marked regression after a single intratumoral reovirus injection. The susceptibility of primary canine MCT cells towards reovirus further supported our hypothesis. We also discovered that normal mast cells derived from bone marrow of dogs and mice are susceptible to reovirus.

Up till now, the key mechanism that determines reovirus susceptibility has not yet been ascertained. Previous report suggests that the Ras signaling pathway plays a vital role in enhancing reovirus disassembly, infectivity and release of progeny virus [28]. Our data, however, strongly suggests that the susceptibility of MCT cell lines does not correlate with the level of activated Ras expression. This was evident in the three canine MCT cell lines that were the most susceptible to reovirus but did not have any activated Ras expression. On the other hand, reovirus did not have such a dramatic effect on HRMC and P815 even though they expressed a high level of activated Ras. Even though a concrete conclusion could not be made due to the limited number of cell lines examined, it is possible that there is a negative correlation between Ras activation and reovirus susceptibility. This negative correlation was also suggested by Twigger *et al*., where the oncolytic effects of reovirus was shown to be independent of the epidermal growth factor receptor (EGFR)/ Ras signaling pathway in head and neck cancer cell lines [13]. Furthermore, the lack of association between reovirus susceptibility and the Ras signaling pathway has also been reported in human hematopoietic cancers [30], colon cancer [31] and non-small cell lung cancer [32]. Moreover, it is known that the *ras* mutation in canine tumors is rare [16], [17]. Thus, it was not surprising that three out of four canine MCT cell lines that were susceptible to reovirus did not have an activated Ras signaling pathway.

Research on reovirus over the past decade have shown that it is very unlikely that the susceptibility of tumor cells towards reovirus lies solely on the simple generic activation of Ras [33]–[39]. Reovirus triggers apoptosis in a caspase-dependent manner [40], which is also reported in this study. Other reports have indicated that reovirus induces apoptosis via the activation of cellular stress kinase, c-Jun N-terminal kinase and nuclear factor kappa B [40], [41]. Hence, there is a possibility that one of these pathways might be the key determinant of reovirus susceptibility.

So far, KIT, a receptor tyrosine kinase, has been the best-described molecular abnormality in canine MCT [15]. Normally expressed on cells such as hematopoietic stem cells, melanocytes and mast cells, KIT regulates apoptosis, cell differentiation, proliferation, chemotaxis and cell adhesion after being activated by stem cell factor [42]. KIT has also been reported to play an equally important role in various human cancers [43]. All of the canine MCT cell lines used in this study have aberrant autophosphorylation of the c-Kit receptor where VIMC, CoMS and CM-MC have KIT mutations and HRMC has a SCF/c-Kit receptor autocrine mechanism [44]. Since all the canine MCT cell lines are susceptible to reovirus, we speculated that the aberrant autophosphorylation of the c-Kit receptor might somehow be involved. However, from this study, we discovered that normal canine and murine mast cells are susceptible to reovirus. Therefore, it is unlikely that the c-Kit receptor contributes to reovirus-induced cell death.

On top of that, the finding that normal mast cells are susceptible to reovirus contradicts the belief that oncolytic viruses only infect and kill cancerous cells while leaving normal cells unharmed in a selective fashion. In a recent report [45], normal mature cells, mainly the peripheral blood mononuclear cells (PBMCs), granulocytes and platelets, were found to be involved in reovirus delivery to the target cancer cells . Normal cells, such as dendritic cells, have also been cited to play an important role in transportation of reovirus to the target tumor cells in order to evade the antiviral immune response [46]. However, this phenomenon does not involve reovirus replication in the dendritic cells [47]. In our study, reovirus replication occurred in normal mast cells but the mechanisms remain unknown. Nonetheless, there is no data to suggest that reovirus poses a significant danger to dogs or other mammals. The safety implications and virus shedding after intentional reovirus infection in dogs are currently under investigation.

The host immune system is an important factor that will dictate the outcome of virotherapy. Unfortunately, unlike in the human population, limited data is available regarding reovirus infection and antibody production in dogs [48]–[50]. Therefore, prediction of the effects of pre-existing neutralizing antibodies in reovirus therapy in dogs is difficult. Immunocompromised mouse models were also used in this study and therapeutic response might be different in immunocompetent hosts. However, the efficacy of oncolytic virotherapy in immunocompetent models has been well documented [51]. Instead of limiting therapy, reovirus have been reported to stimulate the innate and adaptive immune response against tumor cells, acting as immunotherapy to amplify the anti-tumor effects [33], [47], [51]. Nevertheless, the correlation between these laboratory results and the therapeutic efficacy of reovirus in a clinical setting has yet to be established. Therefore, clinical trials in canine MCT patients are necessary to establish the efficacy of reovirus in canine cancers and to evaluate the extent of immune system involvement.

## Conclusion

The feasibility of reovirus therapy in canine MCT is evident with the *in vitro*, *in vivo* and *ex vivo* data obtained in this study. However, there remains a need to clarify the mechanism behind the cytotoxic effects of reovirus in canine MCT as blind usage of reovirus could have disastrous consequences. In summary, as in human cancers, reovirus has great potential as a next generation therapeutic option in veterinary oncology.
